# PAR2 Deficiency Induces Mitochondrial ROS Generation and Dysfunctions, Leading to the Inhibition of Adipocyte Differentiation

**DOI:** 10.1155/2021/6683033

**Published:** 2021-06-08

**Authors:** Yeo Jin Park, Bonggi Lee, Dae Hyun Kim, Eun-Bin Kwon, Younghoon Go, Sugyeong Ha, Min-Kyeong Lee, Hak Sun Yu, Hae Young Chung

**Affiliations:** ^1^Department of Pharmacy, College of Pharmacy, Pusan National University, Busan 46241, Republic of Korea; ^2^Korean Medicine (KM)-Application Center, Korea Institute of Oriental Medicine (KIOM), Daegu 41062, Republic of Korea; ^3^Korean Convergence Medicine, University of Science and Technology, Daejeon 34054, Republic of Korea; ^4^Department of Food Science and Nutrition, Pukyong National University, Daeyeon-dong, Busan, Republic of Korea; ^5^Department of Parasitology and Tropical Medicine, School of Medicine, Pusan National University, Yangsan 50612, Republic of Korea

## Abstract

Protease-activated receptor 2 (PAR2) is a member of G-protein-coupled receptors and affects ligand-modulated calcium signaling. Although PAR2 signaling promotes obesity and adipose tissue inflammation in high fat- (HF-) fed conditions, its role in adipocyte differentiation under nonobesogenic conditions needs to be elucidated. Here, we used several tissues and primary-cultured adipocytes of mice lacking PAR2 to study its role in the development of adipose tissues. C57BL/6J mice with PAR2 deficiency exhibited a mild lipodystrophy-like phenotype in a chow diet-fed condition. When adipocyte differentiation was examined using primary-cultured preadipocytes, PAR2 deficiency led to a notable decrease in adipocyte differentiation and related protein expression, and PAR2 agonist treatment elevated adipocyte differentiation. Regarding the mechanism, PAR2-deficient preadipocytes exhibited impaired mitochondrial energy consumption. Further studies indicated that calcium-related signaling pathways for mitochondrial biogenesis are disrupted in the adipose tissues of PAR2-deficient mice and PAR2-deficient preadipocytes. Also, a PAR2 antagonist elevated mitochondrial reactive oxygen species and reduced the MitoTracker fluorescent signal in preadipocytes. Our studies revealed that PAR2 is important for the development of adipose tissue under basal conditions through the regulation of mitochondrial biogenesis and adipocyte differentiation.

## 1. Introduction

Adipocytes store excess energy as triglycerides (TGs) and act as a dynamic autocrine, paracrine, and endocrine organ that releases hormonal factors. Although excessive growth of white adipose tissue (1) by hyperplasia and hypertrophy can lead to obesity, disruption of adipocyte differentiation also results in metabolic syndrome via ectopic lipid accumulation and lipotoxicity. For instance, genetically and acquired lipodystrophy patients exhibit insulin resistance, hypertriglyceridemia, and nonalcoholic fatty liver diseases (2, 3). Furthermore, various animal studies showed that healthy fat accumulation elevated body weight but greatly improved metabolic syndrome. Mice lacking leptin, while overexpressing adiponectin, exhibited increased expression levels of peroxisome proliferator-activated receptor *γ* (PPAR*γ*), a master transcription factor of adipogenesis, leading to the expansion of adipose tissue; however, these mice showed an improvement in the metabolic syndrome (4). Furthermore, PPAR*γ* agonists improve insulin resistance and dyslipidemia, despite the increase in fat mass. Thus, a supply of fresh adipocytes is essential to maintain energy homeostasis.

Although there are various transcription factors for adipocyte development and functions (5, 6), mitochondria and undifferentiated mesenchymal stem cells (MSCs) are also essential factors for the regulation of metabolic activity in differentiated cells (7, 8). Studies indicate that mitochondria biogenesis and function are essential for adipocyte differentiation. Mitochondrial biogenesis and oxygen consumption elevate notably during adipocyte differentiation, and disruption of these processes inhibits adipocyte differentiation from MSCs (9). When immortalized human MSCs are infected with an adenovirus carrying proliferator-activated receptor *γ* coactivator 1-*α* (PGC-1*α*), a key factor for mitochondrial biogenesis, the cells were suppressed to differentiate into osteoblasts under osteogenic induction and stimulated to differentiate into brown-like adipocytes by upregulation of genes associated with mitochondrial function and lipid metabolism (10). On the other hand, PGC-1*α* knockdown suppressed adipocyte differentiation of MSCs (10). Thus, mitochondrial regulatory processes are mandatory for bioenergetic changes during adipocyte differentiation.

Protease-activated receptor 2 (PAR2) belongs to a family of G-protein-coupled receptors and affects ligand-modulated calcium signaling (11). Although PAR2 signaling is closely associated with obesity and metabolic syndrome under high fat- (HF-) diet-fed conditions, it is also involved in other cellular processes including muscle cell proliferation, skeletal growth, bone repair (12, 13), and mitochondrial activity (14). Because both calcium signaling and PAR2 signaling are associated with mitochondrial functions (14, 15), we examined the roles of PAR2 in adipocyte differentiation using both *in vitro* and *in vivo* analyses.

## 2. Methods and Materials

### 2.1. Materials

SLIGRL-NH_2_ was purchased from Cayman Chemical (Ann Arbor, MI, USA). GB83 was purchased from Axon Medchem (Groningen, The Netherlands). Primary and secondary antibodies were acquired from Abcam (Cambridge, UK), GeneTex (Irvine, CA, USA), Santa Cruz Biotechnology (Dallas, Texas, USA), and Thermo Fisher Scientific (Waltham, MA, USA) (Supplementary Table 1). PVDF membranes were purchased from Millipore Corporation (Bedford, MA, USA). The Dokdo-MARK™ protein size marker was purchased from Elpis Biotech (Daejeon, Korea). Sterile plastic plates for cell culture were purchased from SPL (Seoul, Korea). All other reagents were purchased from Merck (Kenilworth, NJ, USA).

### 2.2. Animals

Five-week-old male WT (C57BL/6J) mice (Daehan Biolink, Seoul, Korea) and PAR2 KO (B6.Cg-F2rl1^tm1Mslb^/J) mice (Provided by Prof. Hak Sun Yu, Department of Parasitology, Pusan National University School of Medicine, Yangsan, Korea) were used in all experiments. All mice were maintained at 23 ± 2°C with a relative humidity of 60 ± 5% and a 12 h/12 h light/dark cycle and provided an unrestricted amount of food and water. Mouse genotyping was performed based on the slightly modified protocol provided by the Jackson Laboratory (Jackson Laboratory, Bar Harbor, ME) (Supplementary Fig. 1). The QuickExtract™ DNA Extraction Solution 1.0 (Epicentre, Wisconsin, USA) was used to extract DNA from mouse tails. Standard PCR was performed with nTaq (Mg^2+^ plus) DNA polymerase (Enzynomics, Daejeon, Korea) and primers. The specific primer sequences to detect PAR2 KO mice were as follows: mutant, 5′-GCC AGA GGC CAC TTG TGT AG-3′; wild type forward, 5′-TCA AAG ACT GCT GGT GGT TG-3′; and wild type reverse, 5′-GGT CCA ACA GTA AGG CTG CT-3′. PCR-derived products were separated on a 1.5% agarose gel and photographed under UV light. Tissues were isolated and frozen instantly in liquid nitrogen for analysis including qPCR, western blot, and other biochemical analyses. We followed the animal protocol approved by the Pusan National University-Institutional Animal Care and Use Committee (PNU-IACUC, Approval No. PNU-2017-1699).

### 2.3. Primary Adipocyte Culture

Subcutaneous adipose tissue was isolated using a midline ventral incision through the skin of 5-week-old male and female mice. The adipose tissue was minced and incubated in Dulbecco's modified Eagle's medium (DMEM) with collagenase type 1 buffer (filter (0.2 *μ*m)-sterilized HEPES buffer including 1 mg/mL collagenase) at 37°C for 90 min with uniform shaking. When the digestion was completed, samples were diluted at a 1 : 1 ratio in 10% FBS/DMEM and processed by filtration using 70 *μ*m cell strainers to remove undigested tissues. Cells were centrifuged at 500 g for 15 min, and the upper lipid layer and supernatant were removed by suction. Sediments were resuspended in 10 mL of red blood cell lysis buffer (BioLegend, San Diego, CA, USA). After incubation at room temperature (22°C) for 10 min, an equal volume of DMEM (10% FBS) was added to the samples. To discard endothelial cell clumps, the cells were filtered again using 40 *μ*m cell strainers, followed by centrifugation at 500 g for 5 min to obtain primary preadipocytes. After the first subculture, cells were plated in 6-well plates or 100*mm* dishes with DMEM (10% FBS) at a density of 2.0 × 10^5^ or 7.0 × 10^5^. Two days after the confluence of the cells, adipocyte differentiation was induced by the addition of a differentiation cocktail (1 *μ*M dexamethasone, 0.5 mM IBMX, 10 *μ*g/mL insulin, and 1 *μ*M rosiglitazone in DMEM supplemented with 10% FBS). After incubation for 48 h, the culture medium was changed with a fresh maintenance medium (DMEM with 10% FBS and 10 *μ*g/mL insulin) every 2 days until day 7. Preadipocytes or fully differentiated adipocytes were used for subsequent experiments. Adipocytes were homogenized to extract RNA and protein or fixed in neutral-buffered formalin for staining.

### 2.4. Histological Analysis

Adipose tissues (eWAT and sWAT) were fixed with neutral-buffered 10% formalin for histochemical examination. Samples were sectioned by paraffin embedding, and hematoxylin and eosin staining was carried out. The Oil Red O staining was performed to visualize lipid accumulation. Differentiated adipocytes were fixed with 10% formalin for 1 h and washed with 60% isopropyl alcohol. Oil Red O solution was treated to the cells for 30 min and rinsed with distilled water. After the staining, images of lipid accumulation were detected with an Olympus IX71 microscope (Olympus, Tokyo, Japan). The dye was eluted with isopropyl alcohol and measured at 510 nm using SpectraMax i3 (Molecular Devices, San Jose, CA, USA).

### 2.5. Western Blot Analysis

Nuclear, cytosolic, and total protein extraction samples of the cells were boiled for 5 min in a loading buffer including 0.2% bromophenol blue, 125 mM Tris-HCl, 10% 2-mercaptoethanol, pH 6.8, and 4% sodium dodecyl sulfate. The protein mixture for each sample was separated by SDS-polyacrylamide gel electrophoresis in 6%–15% acrylamide gels. Subsequently, the separated bands were transferred to PVDF membranes using a Bio-Rad western system (Bio-Rad, Hercules, CA, USA). Protein-transferred membranes were soaked in 5% nonfat milk buffer including 100 mM NaCl, 10 mM Tris (pH 7.5), and 0.1% Tween-20 for 2 h. Then, the membranes were rinsed with TBS-Tween buffer and incubated with primary antibodies at 4°C overnight (Supplementary Table 1). Membranes were rinsed with TBS-Tween buffer for 30 min and incubated with horseradish peroxidase-conjugated secondary antibodies at 25°C for 2 h. The protein bands were visualized by Western Bright Peroxide solution (Advansta, Menlo Park, CA, USA) and enhanced chemiluminescence (Davinch-Chemi CAS400, Seoul, Korea).

### 2.6. Immunocytochemistry

Preadipocytes were plated on a 60 mm cell culture dish and incubated for 24 h. After removing the culture media, cells were incubated with 50 nM MitoTracker Red CMXRos (Thermo Fisher Scientific, Waltham, MA, USA) in serum-free media at 37°C for 20 min. Then, cells were washed with prewarmed PBS and immediately fixed with 4% formaldehyde in distilled water for 30 min. The cells were washed with cold PBS two times and then counterstained with Hoechst 33258 (Thermo Fisher Scientific, Waltham, MA, USA) in PBS (1 : 10000) for 5 min at room temperature in the dark. After washing and mounting, the analysis of images was performed by an FV10i FLUOVIEW Confocal Microscope (Olympus, Tokyo, Japan).

### 2.7. Staining of Mitochondria ROS and Ca^2+^

After coating cover slips with poly-D-lysine (Gibco, USA) in 24 well plates for 15 min, preadipocytes were seeded at a density of 1 × 10^5^ cells/well for 24 h. The cells were treated with drugs at the required concentration (10 *μ*M GB83 and 100 *μ*M SLIGRL-NH_2_) in serum-free media and incubated for 24 h before staining with 1 *μ*M MitoSOX™ Red (Thermo Fisher Scientific, Eugene, OR) or 5 *μ*M Rhod-2 AM (Thermo Fisher Scientific, Eugene, OR) for 30 min at 37°C in the dark. Preadipocytes were washed with PBS, and Hoechst 33342 (Invitrogen, USA) was used to stain the cells. After washing, the cells were observed using a fluorescence microscope (Lionheart FX automated microscope, BioTek, USA). The following quantitative analysis was performed by flow cytometry (cytoFLEX, Beckman Coulter Inc., California, Pasadena, USA).

### 2.8. [Ca^2+^]_i_ Concentration Measurement

Primarily cultured cells were scraped; allowed to sediment at 1,000 g for 5 min; resuspended in a HEPES-buffered medium containing 20 mM HEPES (pH 7.4), 1.2 mM MgSO_4_, 0.5 mM CaCl_2_, 103 mM NaCl, 4.8 mM KCl, 25 mM NaHCO_3_, 15 mM glucose, and 1.2 mM KH_2_PO_4_; and then incubated for 20 min with 5 *μ*M Fura-2-AM. [Ca^2+^]_i_ levels were determined by observing changes in Fura-2 fluorescence (emission wavelength 510 nm and excitation wavelengths 340 nm and 380 nm, every 0.1 sec) using a F4500 fluorescence spectrophotometer (Hitachi, Tokyo, Japan) (Park et al., 2013). Fluorescence intensity ratios (*λ*_340_/*λ*_380_) were used as surrogates of [Ca^2+^]_i_ as described previously (16).

### 2.9. Oxygen Consumption Rate (OCR) Analysis

Cellular OCR was measured using the Seahorse Bioscience XFp Extracellular Flux Analyzer (Seahorse Bioscience, Billerica, MA, USA). Cells were seeded on the XFp cell culture mini plates at 2.0 × 10^4^ cells per well under the various conditions designated in the experiments. The sensor cartridge for the XFp analyzer was hydrated in a 37°C non-CO_2_ incubator overnight. Cells were treated with 1 *μ*M oligomycin (complex V inhibitor), 0.5 *μ*M FCCP, and 0.5 *μ*M rotenone/antimycin A (inhibitors of complex I and complex III) for the OCR test. During sensor calibration, cells were incubated in the 37°C non-CO_2_ incubator in FX running media (4.5 mg/mL d-glucose, 1 mM pyruvate, and 4 mM l-glutamine, pH 7.4) for 1 h. The plate was immediately changed with a sensor cartridge in the calibrated XFp Extracellular Flux Analyzer. OCR was normalized by total protein/well.

### 2.10. Statistical Analysis

Data are expressed as the mean ± standard error of the mean (SEM) unless otherwise indicated. GraphPad Prism 5.0 (GraphPad Software, San Diego, CA, USA) was used for Student's *t*-tests (two-tailed). Differences were considered significant at *P* < 0.05.

## 3. Results

### 3.1. Adipose Tissue Development Is Disrupted in PAR2 KO Mice

**Figure 1:**
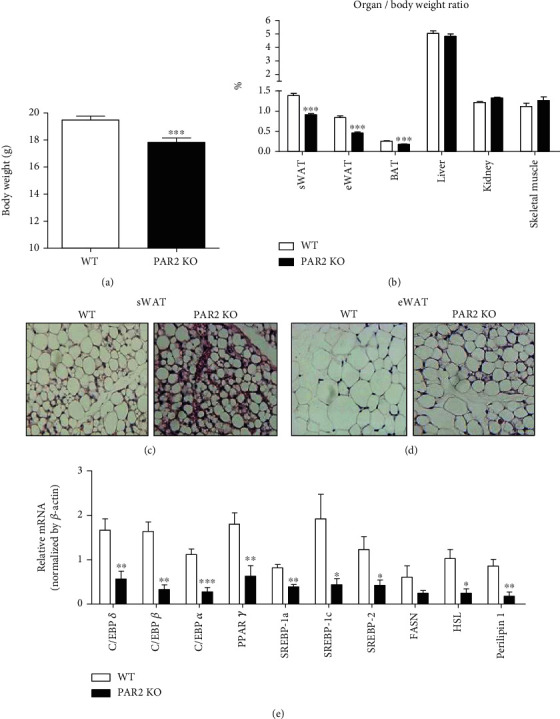
Impairment of adipogenesis in PAR2 KO mice. Tissues were isolated from 5-week-old WT and PAR2 KO male mice. (a) Body weight of WT and PAR2 KO mice (*n* = 31). (b) The organ-to-body weight ratio was calculated to observe differences in the development of tissues between WT and PAR2 KO mice. Representative H&E staining images of (c) sWAT and (d) eWAT of WT and PAR2 KO mice (*n* = 3/group). (e) qPCR was performed to measure mRNA expression levels of adipogenesis and lipolysis markers in sWAT (*n* = 6/group). Data are expressed as the mean ± SEM. ^∗^*P* < 0.05, ^∗∗^*P* < 0.01, and ^∗∗∗^*P* < 0.001 compared to WT.

Although PAR2 signaling is involved in obesity and metabolic syndrome under a HF-fed condition, PAR2 signaling is important not only for cell differentiation and tissue regeneration (17, 18) but also for preventing apoptosis (17, 19). Because it is unclear whether PAR2 signaling affects adipose tissue development under nonobesogenic conditions, we investigated the effects of PAR2 deficiency on energy balance and adipose tissue development under a chow diet-fed condition. When body weight was measured at an early age (5 weeks), it was significantly decreased in PAR2 KO mice compared to WT mice fed a chow diet (Figure 1(a)). There were no differences in the percent weight of the liver, kidney, and skeletal muscle, but the percentage of subcutaneous fat (sWAT), epididymal fat (eWAT), and brown fat (BAT) was markedly reduced in PAR2 KO mice (Figure 1(b)), indicating that the development of adipose tissue was abnormal under PAR2-deficient conditions. To further examine this, H&E staining was performed. Interestingly, sWAT and eWAT of PAR2 KO mice were not filled with large, closely packed adipocytes compared to those of WT mice; this characteristic is more apparent for sWAT (Figures 1(c) and 1(d)). Thus, we tested whether PAR2 deficiency is associated with adipocyte differentiation by measuring mRNA levels of adipocyte differentiation markers in sWAT. Data from quantitative PCR (qPCR) showed that mRNA levels of adipogenic and lipogenic transcription factors including CCAAT/enhancer-binding protein *α*/*β*/*δ* (CEBP*α*/*β*/*δ*), PPAR*γ*, *sterol regulatory element-binding protein*-1c (SREBP1c), and SREBP2 are notably decreased in sWAT of PAR2 KO mice (Figure 1(e)). Consistently, mRNA levels of their downstream genes including fatty acid synthase (FASN), hormone-sensitive lipase (HSL), and perilipin 1 were also significantly decreased (Figure 1(e)). These data suggest that the development of adipose tissues is disrupted in PAR2 KO mice.

### 3.2. PAR2 Deficiency Leads to a Defect in Adipocyte Differentiation

**Figure 2:**
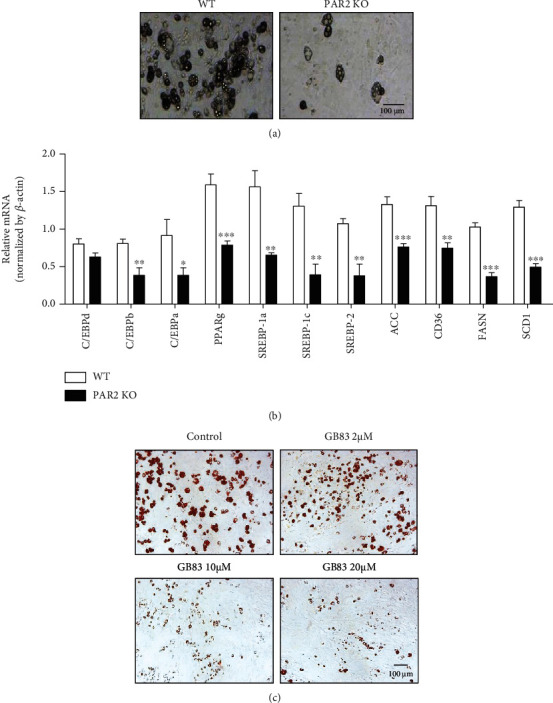
PAR2 deficiency impairs adipocyte differentiation. Preadipocytes were isolated from sWAT of male WT and PAR2 KO mice (aged 5 weeks). Cells were differentiated into adipocytes for 7 days. (a) Representative microscopic images of primary-cultured adipocytes are shown. (b) qPCR was performed using primary-cultured adipocytes to measure mRNA expression levels of genes associated with adipocyte differentiation (*n* = 6/group). (c) Oil Red O staining images of primary-cultured adipocytes with or without GB83 treatment (*n* = 4). Data are expressed as the mean ± SEM. ^∗^*P* < 0.05, ^∗∗^*P* < 0.01, and ^∗∗∗^*P* < 0.001 compared to WT.

**Figure 3:**
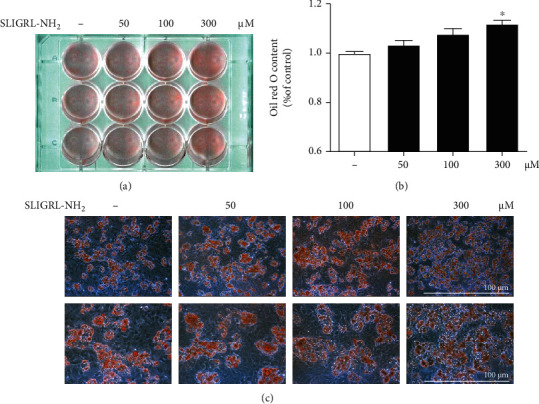
PAR2 agonist increases preadipocyte differentiation into adipocytes. Preadipocytes were isolated from sWAT of male WT mice (aged 5 weeks). Preadipocytes were treated with the PAR2 agonist (SLIGRL-NH_2_) and differentiated into adipocytes for 7 days followed by Oil Red O staining. (a) Visible images of stained adipocytes. (b) Oil Red O was extracted with isopropanol, and the absorbance of the extracted dye was measured at 510 nm. (c) Oil Red O staining images of primary-cultured adipocytes with or without SLIGRL-NH_2_ treatment (*n* = 3). Data are expressed as the mean ± SEM. ^∗^*P* < 0.05 compared to the control group.

To test whether the decrease in adipose tissue development in PAR2 KO mice was due to a cell-autonomous effect, preadipocytes were isolated from sWAT of both WT and PAR2 KO mice and differentiated into adipocytes for 7 days. WT preadipocytes differentiated well into adipocytes as determined by the microscopic analysis, whereas PAR2 KO preadipocytes showed a defect in adipocyte differentiation (Figure 2(a)). When comparing between male and female mice, the decrease in adipocyte differentiation is more apparent in male PAR2-KO mice (Supplementary Fig. 2). We found that the defect in the differentiation ability occurred at a very early stage of the differentiation process (day 1-2 after the differentiation cocktail treatment, unpublished data). When mRNA levels of transcription factors related to adipogenesis were tested by qPCR, CEBP*α*, CEBP*β*, PPAR*γ*, SREBP1a, SREBP1c, and SREBP2 mRNA levels were significantly decreased in PAR2 KO adipocytes (Figure 2(b)). Correspondingly, mRNA levels of the downstream target genes ACC, CD36, FASN, and SCD1 were also reduced (Figure 2(b)). We next investigated whether treatment with GB83, a PAR2 antagonist, affected the differentiation of preadipocytes isolated from WT mice. The cytotoxicity assay showed that GB83 had no toxicity up to 50 *μ*M in preadipocytes (Supplementary Fig. 3). Oil Red O staining showed that adipocyte differentiation was suppressed in a concentration-dependent manner when WT preadipocytes were treated with GB83 during the differentiation process (Figure 2(c)). To further confirm the PAR2-mediated regulation of adipocyte differentiation, we treated WT preadipocytes with a PAR2 agonist (SLIGRL-NH_2_) at various concentrations and Oil Red O staining was performed. The images and quantified data from Oil Red O staining exhibit that PAR2 activation elevates preadipocyte differentiation (Figures 3(a)–3(c)). These data indicate that PAR2 signaling is necessary for preadipocyte differentiation into adipocytes, and the effect appears to be cell autonomous.

### 3.3. PAR2-Deficient Adipocytes Have a Defect in Mitochondrial Biogenesis and Energy Expenditure

**Figure 4:**
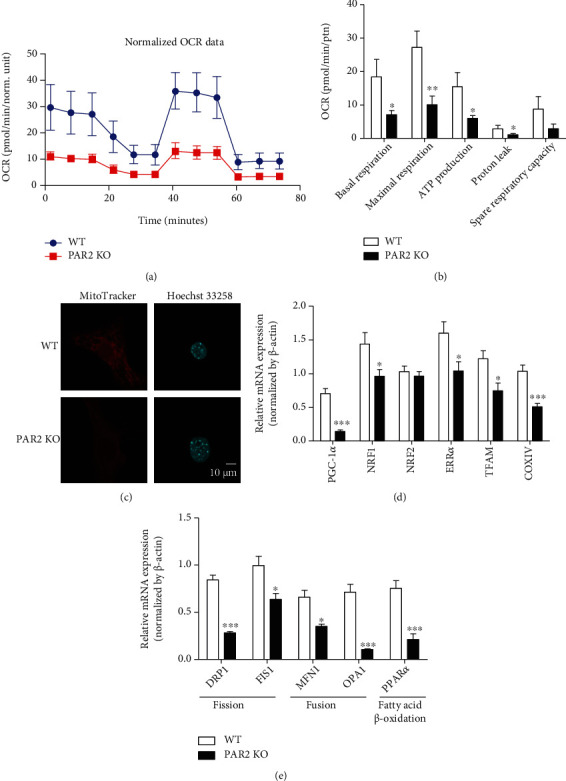
PAR2 deficiency decreases mitochondrial biogenesis and mitochondrial function. Preadipocytes were isolated from sWAT of male WT and PAR2 KO mice (aged 5 weeks). (a) Oxygen consumption rate (OCR) was measured in primary-cultured preadipocytes using the Seahorse XFp analyzer (*n* = 3). After recording thrice the baseline for OCR measurements, oligomycin (Oligo), FCCP, rotenone (Rot), and antimycin A (Anti) were added to the cells and OCR measurements were taken three times after each drug treatment. (b) Quantification of parameters of mitochondrial OCR analyzed by the Seahorse XFp analyzer (*n* = 3). These experiments were repeated three times. (c) Representative staining images of MitoTracker and Hoechst 33258 in preadipocytes. To measure mRNA levels of genes related to mitochondrial biogenesis and mitochondrial function in adipocytes, preadipocytes were isolated from sWAT of male WT and PAR2 KO mice (aged 5 weeks) and differentiated to adipocytes for 7 days. The mRNA expression level of genes associated with (d) mitochondrial biogenesis and (e) mitochondrial function. Data are expressed as the mean ± SEM. ^∗^*P* < 0.05 and ^∗∗∗^*P* < 0.001 compared to WT.

The process of adipocyte differentiation requires a large amount of energy when cells become fully metabolically active (20). Because we observed that the defect in adipocyte differentiation of PAR2-deficient preadipocytes occurred at a very early stage, we investigated the energy metabolism of PAR2-deficient preadipocytes using the Seahorse XF analyzer. Specifically, the mitochondrial oxygen consumption rate (OCR) was measured in the primary cultureed preadipocytes isolated from sWAT of WT and PAR2 KO mice under serum-free conditions. PAR2 KO preadipocytes had substantially lower basal and maximal cellular respiratory capacities than WT preadipocytes (Figures 4(a) and 4(b)). Similarly, there was a significant decrease in the ATP synthesis capacity and proton leak in PAR2 KO preadipocytes (Figure 4(b)). Spare respiratory capacity also trended lower in PAR2 KO preadipocytes (Figure 4(b)). In addition, the MitoTracker fluorescent signal was weaker in PAR2 KO preadipocytes than in WT preadipocytes (Figure 4(c)). These data indicate that mitochondrial dysfunction occurs in PAR2-deficient preadipocytes. We next investigated whether the dysfunction in mitochondria occurs in adipocytes differentiated from PAR2 KO sWAT by measuring mRNA levels of mitochondria-related markers. The mRNA levels of mitochondrial biogenesis markers including PGC-1*α*, NRF1, NRF2, ERR*α*, TFAM, and COX IV were reduced in PAR2-deficient adipocytes (Figure 4(d)). In addition, PAR2 deficiency downregulated mRNA levels of mitochondria fusion genes including mitofusin 1 (MFN1) and optic atrophy 1 (OPA1) as well as mitochondria fission genes including dynamin-related protein 1 (DRP1) and mitochondrial fission 1 protein (FIS1) (Figure 4(e)). Moreover, deficiency of PAR2 significantly attenuated mRNA expression of PPAR*α*, a *β*-oxidation-related gene (Figure 4(e)). These results indicated that PAR2 plays an important role in mitochondrial biogenesis and mitochondrial function of primary-cultured preadipocytes and adipocytes.

### 3.4. sWAT of PAR2 KO Mice Has Reduced Mitochondria Number and Altered Expression Profile of Genes Related to Mitochondrial Function

**Figure 5:**
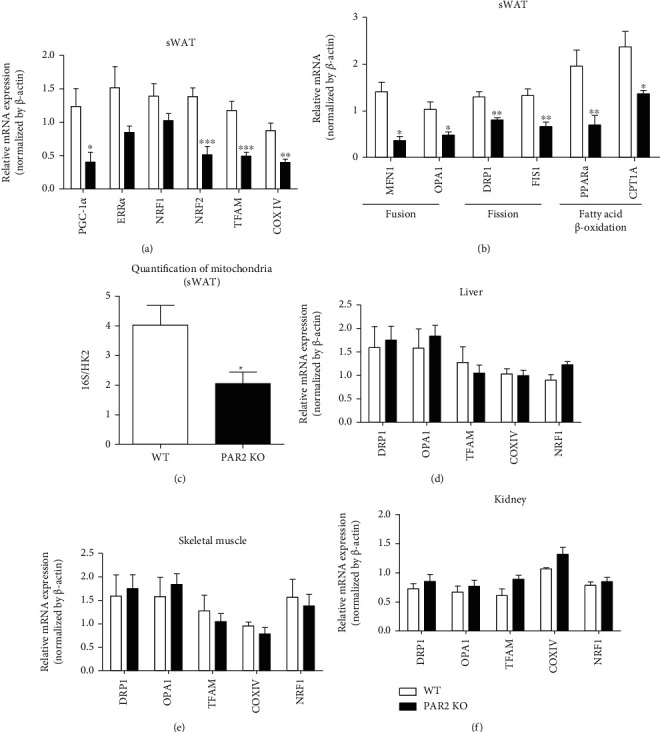
Expression profile of mitochondria-related genes is changed only in adipose tissues of PAR2 KO mice. Tissues were isolated from male WT and PAR2 KO mice (aged 5 weeks). The mRNA expression levels of mitochondrial genes were analyzed in the (45) sWAT, (d) liver, (e) skeletal muscle, and (f) kidney tissues by qPCR. (c) Mitochondrial DNA was quantified in sWAT by qPCR to predict the number of mitochondria. Data are expressed as the mean ± SEM. ^∗^*P* < 0.05, ^∗∗^*P* < 0.01, and ^∗∗∗^*P* < 0.001 compared to WT.

We further tested whether the dysfunction in mitochondria occurs in tissues of PAR2 KO mice by measuring mRNA levels of mitochondria-related genes or by quantifying mitochondrial DNA. The mRNA levels of mitochondrial biogenesis markers including PGC-1*α*, NRF2, TFAM, and COX IV were reduced in the sWAT of PAR2 KO mice (Figure 5(a)). The mRNA levels of ERR*α* and NRF1 trended lower in PAR2 KO sWAT (Figure 5(a)). In addition, PAR2 deficiency downregulated mRNA levels of mitochondria fusion genes including MFN1 and OPA1 as well as mitochondria fission genes including DRP1 and FIS1 in sWAT (Figure 5(b)). When mRNA levels of fatty acid oxidation-related genes were measured, PPAR*α* and CPT1A were downregulated in PAR2 KO sWAT (Figure 5(b)). Consistent with the gene expression profile, the mitochondria over nuclear DNA ratio (mtDNA/nDNA, 16S/HK2) was significantly decreased in the sWAT of PAR2 KO mice compared to that of WT mice (Figure 5(c)). However, there were no differences in mRNA levels of mitochondria-related genes in the liver, skeletal muscle, and kidney between WT and PAR2 KO mice (Figures 5(d)–5(f)). Together, these results suggest that PAR2 is involved in mitochondrial biogenesis and mitochondrial function in adipose tissues.

### 3.5. PAR2 Is Associated with Calcium-Mediated Mitochondrial Biogenesis Pathways

**Figure 6:**
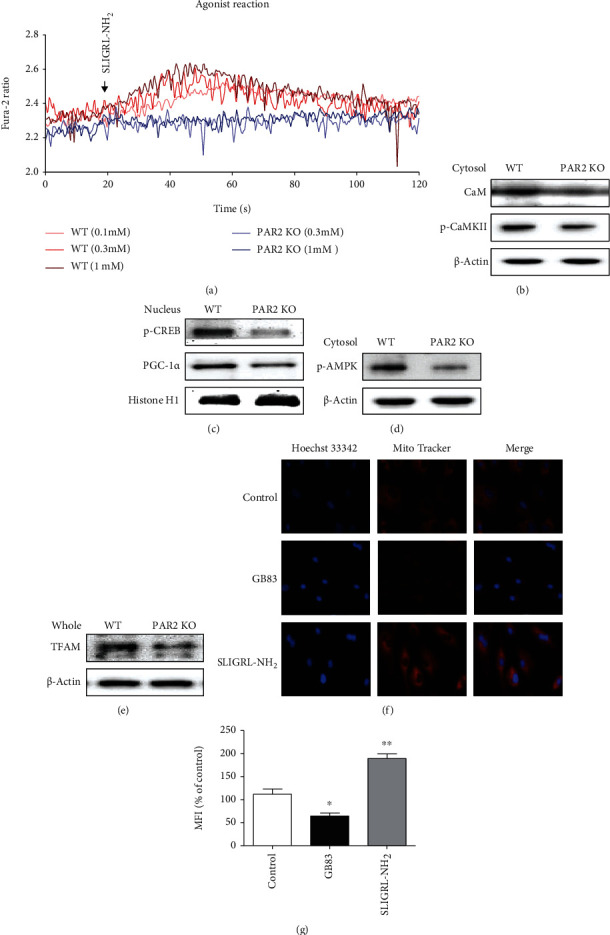
PAR2 deficiency reduces calcium signaling in preadipocytes. Preadipocytes were isolated from sWAT of male WT and PAR2 KO mice (aged 5 weeks). (a) SLIGRL-NH_2_-mediated calcium mobilization by activation of PAR2. Representative images of western blot analysis show protein levels of (b) CaM and p-CaMKII (cytosol), (c) p-CREB and PGC-1*α* (nucleus), (d) p-AMPK (cytosol), and (e) TFAM (whole lysate) in the cells. (f) Preadipocytes were treated with the PAR2 agonist (SLIGRL-NH_2_) or antagonist (GB83) for 24 h, and fluorescent microscopic analysis was performed. (g) MitoTracker staining images were quantified by ImageJ (*n* = 3). Data are expressed as the mean ± SEM. ^∗^*P* < 0.05 and ^∗∗^*P* < 0.01 relative to control.

Studies showed that PAR2 activation stimulates endogenous calcium channels to adjust intracellular calcium mobilization (21–23). In addition, it has been reported that intracellular calcium signaling is necessary for mitochondrial biogenesis (24, 25). To determine whether PAR2 also increases intracellular calcium concentrations in preadipocytes, the PAR2-specific agonist, SLIGRL-NH_2_, and Fura-2/AM, a high-affinity Ca^2+^ selective fluorescent indicator, were used. The PAR2 agonist induced a concentration-dependent calcium response in cells that express PAR2 (Figure 6(a)). However, no response was found in PAR2-deficient preadipocytes (Figure 6(a)), indicating that PAR2 is important for intracellular calcium signaling.

Ca^2+^ is a second messenger that regulates various cell functions by making a complex with calmodulin (CaM), a pervasive intracellular Ca^2+^ receptor. Combining Ca^2+^ with CaM activates several CaM-binding proteins including CaM kinases (CaMKI, CaMKII, and CaMKIV) (26). Of these, CaMKII is associated with the activation of CREB and AMPK, which are important factors for the induction of PGC-1*α*, a central regulator for mitochondrial biogenesis (27–29). We tested the relationship between PAR2 and the signaling pathways associated with calcium-related mitochondrial biogenesis in preadipocytes. Western blotting showed that PAR2-deficient preadipocytes had reduced protein levels of CaM and CaMKII in the cytosol compared to those of WT preadipocytes (Figure 6(b)). Protein levels of key regulators of mitochondrial biogenesis including p-CREB and PGC-1*α* were also decreased in the nucleus of PAR2-deficient preadipocytes (Figure 6(c)). Furthermore, protein levels of p-AMPK, another mitochondrial biogenesis-related protein regulated by calcium signaling, were reduced in the cytosol of PAR2-deficient preadipocytes (Figure 6(d)). Consistently, protein levels of TFAM, a mitochondrial biogenesis marker, were decreased in PAR2-deficient preadipocytes (Figure 6(e)). In addition, the MitoTracker fluorescent signal is weaker in preadipocyte treated with PAR2 antagonist (GB83) and stronger in preadipocyte treated with PAR2 agonist (SLIGRL-NH_2_) than the control group (Figure 6(f)). These data showed that PAR2-mediated calcium signaling is associated with mitochondrial biogenesis and functions in preadipocytes.

### 3.6. PAR2 Is Negatively Related to Mitochondrial ROS Generation

**Figure 7:**
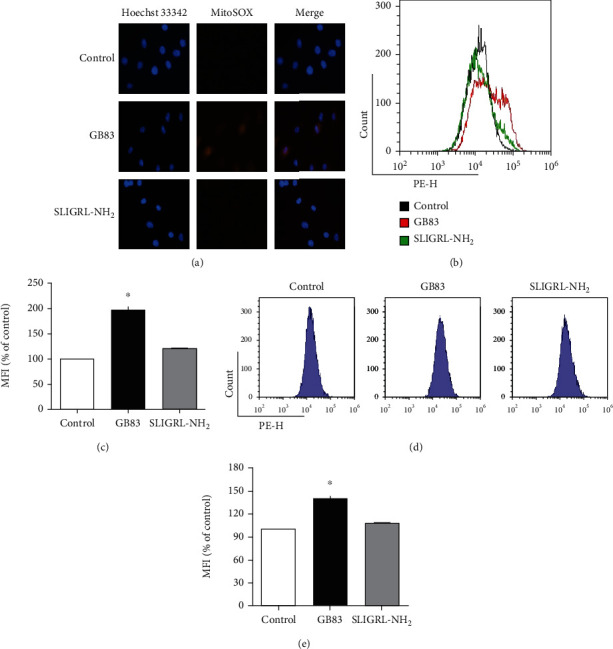
PAR2 regulated mitochondrial ROS and calcium levels in preadipocytes. Preadipocytes were isolated from sWAT of male WT mice (aged 5 weeks) and treated with the PAR2 agonist (SLIGRL-NH_2_) or antagonist (GB83). Mitochondrial ROS levels were observed using mitochondrial superoxide indicator MitoSOX by (a) fluorescent microscopy and (b) histogram of flow cytometry. (c) The data from flow cytometry were quantified. To observe mitochondrial calcium, Rhod-2, Ca^2+^-sensitive dye Rhod-2 to monitor changes in mitochondrial Ca^2+^ was used for (d) flow cytometry. (e) The data from flow cytometry were quantified. Data are expressed as the mean ± SEM. ^∗^*P* < 0.05 compared to the control group.

Mitochondrial calcium overload can induce the generation of reactive oxygen species (ROS), triggering the permeability transition pore and leading to mitochondrial dysfunctions (30). We determined whether PAR2 is associated with mitochondrial ROS generation using MitoSOX, a mitochondrial superoxide indicator, and mitochondrial calcium using Rhod-2, the fluorescent calcium indicator selectively accumulated within mitochondria. The fluorescent images and data from flow cytometry showed that PAR2 antagonist treatment in preadipocyte significantly elevated mitochondrial ROS levels (Figures 7(a)–7(c)). To test the elevated ROS in the PAR2 antagonist-treated group associated with mitochondrial calcium levels, we performed flow cytometry, and the results showed that mitochondrial calcium (Rhod-2) was notably elevated (Figures 7(d) and 7(e)), indicating that the elevated mitochondrial calcium is related to the increase in mitochondrial ROS in preadipocytes treated with the PAR2 antagonist.

## 4. Discussion

Although PAR2 signaling is associated with adipose inflammation, obesity, and metabolic syndrome under a HF-fed condition (31, 32), its role in adipose tissue under nonobesogenic conditions has not been yet examined. Here, we report a previously unrecognized role of PAR2 in adipocyte differentiation. PAR2 deficiency leads to a mild lipodystrophy-like phenotype due to impairment in adipocyte differentiation in chow diet-fed mice. As a potential mechanism, PAR2-deficient preadipocytes exhibit a marked decrease in mitochondrial respiration possibly due to disruption of calcium signaling-mediated mitochondrial biogenesis. Thus, PAR2 signaling may be necessary for the proper development of adipose tissues in nonobesogenic environments.

Mitochondrial biogenesis is mandatory for adipocyte differentiation (9). For example, mitochondrial oxidative metabolism produces ATP for dynamic changes; moreover, mitochondrial anaplerosis is critical for generating glycerol 3-phosphate to synthesize TGs in adipocytes (33, 34). In addition, the generation of acetyl-CoA before the esterification of TGs may need a relatively large number of mitochondria. Moreover, *β*-oxidation of FAs may be a crucial energy source in adipocytes (35). On the other hand, the knockdown of mitochondria fusion genes resulted in the loss of adipocyte differentiation capacity (36). Our data shows that sWAT and PAR2-deficient adipocytes exhibit a decrease in mRNA levels in the genes associated with mitochondrial biogenesis, dynamics, and *β*-oxidation. Furthermore, the reduction in mitochondrial biogenesis signaling was observed in PAR2-deficient preadipocytes. Considering that the impairment in adipocyte differentiation occurs at a very early stage after treatment with the differentiation cocktail, we assume that the impairment in mitochondrial biogenesis and dysfunction in PAR2-deficient preadipocytes greatly contribute to the impaired development of adipose tissues in PAR2 KO mice.

Although mitochondrial biogenesis is inhibited under PAR2-deficient conditions, the underlying mechanisms are unclear. We considered the possibility that PAR2-mediated calcium signaling is related to this process. PAR2 has been shown to activate calcium signaling (37), and phosphorylated CaMKII induces activation of signaling molecules and several other transcription factors including CREB (38). Characterization of the promoter region in the PGC-1*α* gene exhibited a conserved CREB binding sequence (39, 40). Our study also showed that treatment with a PAR2 agonist induced calcium-mediated CaMKII activation followed by CREB phosphorylation in WT preadipocytes, whereas no response was found in PAR2-deficient preadipocytes. CaMKII has also been shown to regulate the activity of AMPK (41). Several insights from different *in vivo* transgenic models demonstrated that AMPK directly affects PGC-1*α* activity through phosphorylation (42, 43). Similarly, our study showed that activation of AMPK was lower in PAR2 KO preadipocytes. Additionally, protein levels of PGC-1*α* in the nucleus were decreased in PAR2 KO preadipocytes. Thus, we assume that PAR2-mediated calcium signaling, followed by the activation of CREB and AMPK, contributes to mitochondrial biogenesis.

A G-protein-coupled receptor 103 (GPR103) has been reported as a regulator of adipocyte metabolism (44). When pyroglutamylated RFamide peptides (QRFPs), endogenous peptide ligands of GPR103, were treated in fully differentiated 3T3-L1 adipocytes, intracellular triglyceride levels were elevated based on Oil Red O staining followed by densitometry-based quantification. These effects were mediated partly due to the lipoprotein lipase-mediated increase in fatty acid uptake and reduced lipolysis after isoproterenol treatment (44). While shRNA-mediated knockdown of GPR103b did not affect adipocyte differentiation, it suppressed QRFP-mediated fatty acid uptake. These data suggest that GPR103b receptor signaling is important for lipid accumulation in differentiated adipocytes (44). Based on our data, the differentiation capacity of PAR2-deficient preadipocytes was lower than that of WT preadipocytes possibly due to impaired calcium-related signaling pathways that lead to mitochondrial biogenesis and mitochondrial oxidative stress. Thus, it appears that the action mechanisms of GPR103b and PAR2 on adipocyte functions look different although further studies are necessary.

In conclusion, PAR2 plays an important role in adipocyte differentiation partly by activating CaMKII/CREB/AMPK signaling, followed by mitochondrial biogenesis. Thus, PAR2 is necessary for maintaining an energy balance by regulating the development of adipose tissue under nonobesogenic conditions.

## Data Availability

Data is available on request.

## Supplementary Materials

Supplementary Materials This paper includes supplementary materials (supplementary figures and table) which are available online.
