# Supplementary material for: PAR2 Deficiency Induces Mitochondrial ROS Generation and Dysfunctions, Leading to the Inhibition of Adipocyte Differentiation
Source: Oxid Med Cell Longev. 2021 Jun 8;2021:6683033. doi: 10.1155/2021/6683033 (PMC8205587; doi:10.1155/2021/6683033)
Supplement: Supplementary Materials — This paper includes supplementary materials (supplementary figures and table) which are available online. [file 6683033.f1.docx]

**Supplementary Figures and tables**


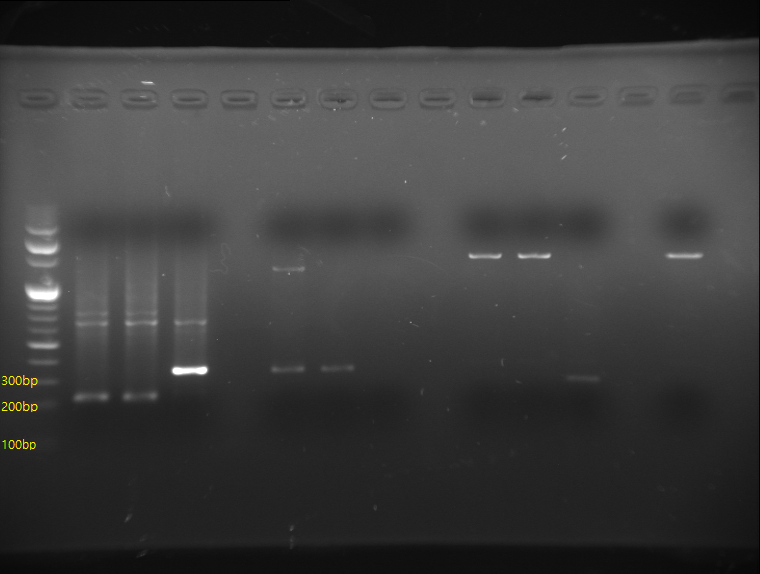

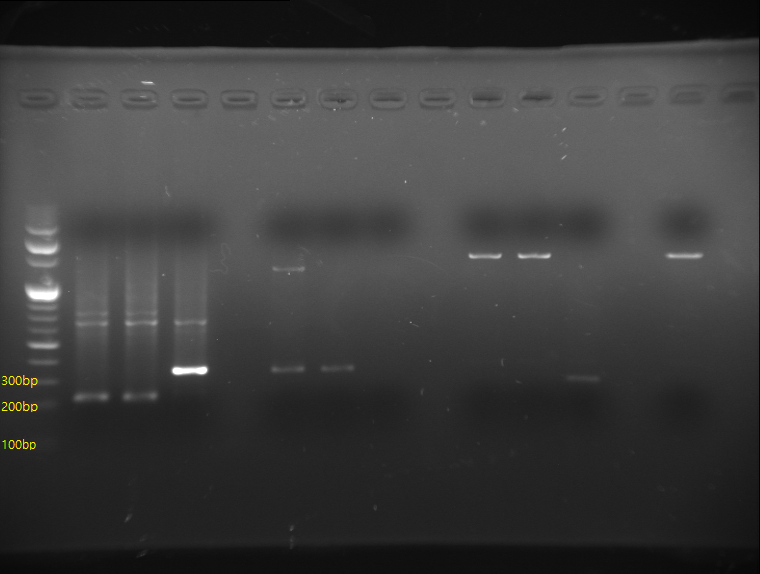

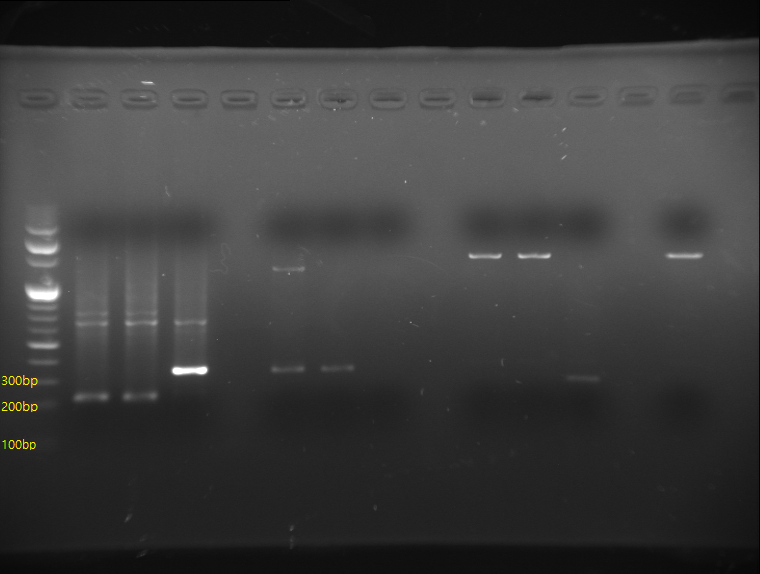


**PAR2 KO**

**WT**

**345bp**

**198bp**

**Supplementary figure 1. The genotyping result of WT and PAR2 KO mice.**

PCR-based genotyping of WT and PAR2 KO mice. Amplified fragments using specific primers reveal the mutant (198 bp) and endogenous (345 bp) PAR2 alleles.


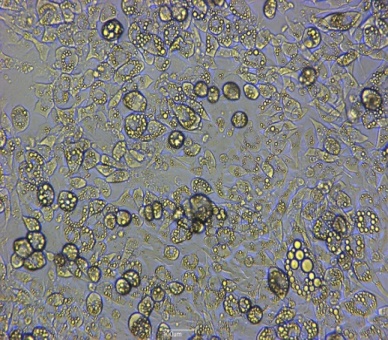

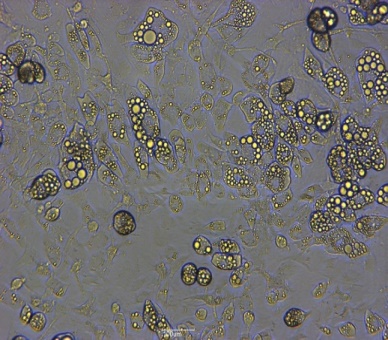

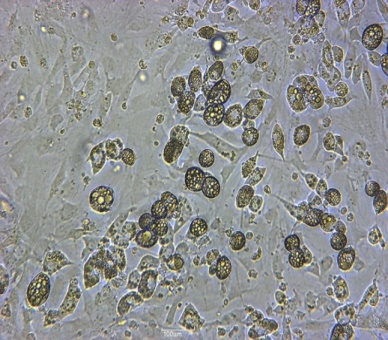

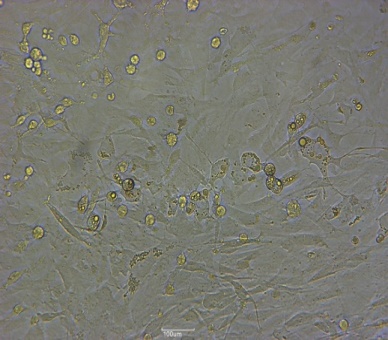


**WT**

**female**

**PAR2KO**

**female**

**WT**

**male**

**PAR2 KO**

**male**

100 μm

**Supplementary figure 2.** The defect in adipocyte differentiation is more apparent in male PAR2 KO mice. Pre-adipocytes were differentiated to adipocytes for 7 days. The images of differentiated adipocytes were shown.

**Supplementary figure 3.** Cell viability assay of GB83 in the primary-cultured preadipocytes. Preadipocytes were isolated from sWAT of C57BL/6J male mice aged 5 weeks. Cell viability was measured in the preadipocytes, which was treated with GB83 dose-dependently for 24 h (n=6).

**Supplementary tables**

**Supplementary table 1.** Primary and secondary antibodies used in experiments

| **Antibody** | **Host animal** | **Dilution** | **Company** |
| --- | --- | --- | --- |
| β-actin | Mouse | 1:1000 | Santa Cruz (sc-47778) |
| Histone H1 | Rabbit | 1:1000 | GeneTex (GTX114462) |
| CaM (FL-149) | Rabbit | 1:1000 | Santa Cruz (sc-5537) |
| PPARγ | Rabbit | 1:1000 | Santa Cruz (sc-7196) |
| SIRT1 | Rabbit | 1:1000 | Abcam (ab13749) |
| PGC-1α | Rabbit | 1:1000 | Santa Cruz (sc-13067) |
| FoxO1 (H-130) | Rabbit | 1:1000 | Santa Cruz (sc-67140) |
| p-CREB-1(Ser133) | Rabbit | 1:1000 | Santa Cruz (sc-101663) |
| AMPK | Rabbit | 1:1000 | Santa Cruz (sc-25792) |
| p-AMPK (Thr172) | Rabbit | 1:1000 | Santa Cruz (sc-33524) |
| p-CaMKⅡα (Thr286) | Rabbit | 1:2000 | Thermo Fisher scientific (MA1-047) |
| [Anti-rabbit IgG-HRP](https://www.scbt.com/scbt/product/mouse-anti-rabbit-igg-hrp?requestFrom=search) | Mouse | 1:10000 | Santa Cruz (sc-2357) |
| [Anti-mouse IgG-HRP](https://www.scbt.com/scbt/product/mouse-anti-rabbit-igg-hrp?requestFrom=search) | Goat | 1:10000 | Santa Cruz (sc-2005) |

**Supplementary table 2.** Primer sequences of qRT-PCR

| **Gene** | **Forward (5’-3’)** | **Reverse (3’-5’)** |
| --- | --- | --- |
| **Mouse** |  |  |
| *ACC* | AGGATCATATGGGGCCTTTG | AGGATCATATGGGGCCTTTG |
| *CD36* | TTGTGGCCTTGCACTCTCTC | TCTCCTCGTGCAGCAGAATC |
| *C/EBP α* | AAACAACGCAACGTGGAGAC | TGTCCAGTTCACGGCTCAG |
| *C/EBPβ* | GGGTTGTTGATGTTTTTGGTTT | GAAACGGAAAAGGTTCTCAAAA |
| *C/EBPδ* | ACTCACAGCAGTCCACAAGC | CAAACCCTCCAGGGTCTAAATAC |
| *COX IV* | CTCCAACGAATGGAAGACAG | TGACAACCTTCTTAGGGAA |
| *DRP1* | CCGACTTTGCTGATGCCTGT | TTGTCCCGTGATCCAGCTGA |
| *ERRα* | CTCAGCTCTCTACCCAAACGC | CCGCTTGGTGATCTCACACTC |
| *FASN* | GAAACCTGACGGCATCATTG | CGGTGTCCTCAGAGTTGTGG |
| *FIS1* | CCCTGCTACTGGACCATGGA | CCACAGACACCAGCTCGTTC |
| *HSL* | TGAGATGGTAACTGTGAGCC | ACTGAGATTGAGGTGCTGTC |
| *MFN1* | TCCCTCACGTCGAGAACCTC | ATCTGCAGCTTCTCGGTTGC |
| *NRF1* | GCACCTTTGGAGAATGTGGT | CTGAGCCTGGGTCATTTTGT |
| *NRF2* | CCAGCTACTCCCAGGTTGC | CCTGATGAGGGGCAGTGA |
| *OPA1* | GCGCTTCAAGGTCGTCTCAA | CTTGCGAGGTAAGCTGGGTG |
| *SREBP2* | GCTCCAGCTCATCAACAACCA | CAGGAAGGCTTCCAGAGAGGA |
| *TFAM* | GAAGGGAATGGGAAAGGTAGA | AACAGGACATGGAAAGCAGAT |
| *β-actin* | AGTGTGACGTTGACATCCGT | TGCTAGGAGCCAGAGCAGTA |
| *Perilipin1* | CTCTGGGAAGCATCGAGAAG | GCATGGTGTGTCGAGAAAGA |
| *PGC-1α* | CCCTGCCATTGTTAAGACC | TGCTGCTGTTCCTGTTTTC |
| *PPARα* | CATGTGAAGGCTGTAAGGGC | TCTTGCAGCTCCGATCACACT |
| *PPARγ* | TCGCTGATGCACTGCCTATG | GAGAGGTCCACAGAGCTGAT |
| *SCD1* | ATGGATATCGCCCCTACGAC | TCTTGTGACTCCCGTCTCCA |
| *SREBP1a* | GCGCCATGGACGAGCTG | TTGGCACCTGGGCTGCT |
| *SREBP1c* | GGAGCCATGGATTGCACATT | GCTTCCAGAGAGGAGGCCAG |
